# The Beneficial Effects of Marine Plant-Derived Compounds on the Musculoskeletal System

**DOI:** 10.3390/ijms27021032

**Published:** 2026-01-20

**Authors:** László Szabó, Áron Gere, Zsigmond Máté Kovács, Tamás Bazsó, Beatrix Dienes

**Affiliations:** 1 Department of Physiology, Faculty of Medicine, University of Debrecen, 4032 Debrecen, Hungary; laszlo.szabo@med.unideb.hu (L.S.); gere.aron@med.unideb.hu (Á.G.); kovacs.zsigmond@med.unideb.hu (Z.M.K.); 2 HUN-REN DE Cell Physiology Research Group, University of Debrecen, 4032 Debrecen, Hungary; 3 Doctoral School of Molecular Medicine, Faculty of Medicine, University of Debrecen, 4032 Debrecen, Hungary; 4 Department of Orthopaedic Surgery and Traumatology, Faculty of Medicine, University of Debrecen, Bartók B. út 2-26, 4031 Debrecen, Hungary; bazso.tamas@med.unideb.hu

**Keywords:** marine plant derivatives, anti-inflammatory effects, musculoskeletal system, muscle atrophy, osteoarthritis

## Abstract

The skeletal muscle system is particularly susceptible to degenerative and inflammatory processes that threaten mobility, quality of life, and systemic health. Marine plants, including brown, red, and green algae, are valuable yet understudied sources of bioactive compounds with therapeutic potential against skeletal muscle inflammation and degeneration. This narrative review provides the first overview of polyphenols, polysaccharides, carotenoids, and multiminerals derived from marine plants, with a particular focus on their effects on skeletal muscle, bone, and joint tissues. It highlights both the therapeutic potential and the limitations of marine plant-derived bioactive compounds in the musculoskeletal system. The compounds discussed, such as phlorotannins, ulvan, fucoidan, carotenoids, spirulina derivatives, and Aquamin, modulate key signaling pathways, including NF-κB, JAK/STAT3, and the NLRP3 inflammasome. Among these, MAPK emerges as the most consistently affected axis across all compound classes, leading to a reduction in TNF-α, IL-1β, IL-6, and oxidative stress markers. These bioactive compounds have been shown in both in vitro and in vivo models to reduce muscle catabolism, enhance osteoblast differentiation and mineralization, and reduce cartilage inflammation. Despite favorable safety, biocompatibility, and biodegradability profiles, current evidence shows that systemic applications significantly dominate over local delivery, highlighting the untapped potential of localized delivery strategies. Overall, this narrative review underscores the growing importance of marine plant-derived bioactives as promising natural agents for maintaining musculoskeletal integrity and alleviating degenerative disorders.

## 1. Introduction

Life is movement, and movement is life. Every day, we can enjoy the benefits of a healthy musculoskeletal system, a complex, closely cooperating network of tissues that is highly sensitive to injury, degeneration, and age-related decline. A sore muscle or knee can significantly diminish quality of life through reduced physical performance. Even a slight inflammation in the musculoskeletal system can have a grave effect on a patient’s health. In aging societies, the prevention and early treatment of inflammatory diseases is an increasingly important sociological and ecological problem. So, it is essential to find inexpensive but reliable methods for treating musculoskeletal inflammation. Marine ecosystems are rich sources of bioactive compounds with various pharmacological properties, offering a potential alternative solution to the problem. Organic compounds derived from seaweed and algae provide essential macro- and micronutrients and are suitable for patients with metabolic risks such as hyperglycemia, hypercholesterolemia, and hyperlipidemia.

Due to the extreme and rapidly changing environmental conditions of marine habitats—such as high salinity, hydrostatic pressure, low temperatures, variable light exposure, and constant mechanical stress—marine plants have evolved a remarkable degree of metabolic plasticity. This adaptation has resulted in the biosynthesis of structurally unique secondary metabolites that are rarely found in terrestrial species [1]. These compounds often exhibit enhanced antioxidant and anti-inflammatory activity, reflecting their ecological role in stress resistance and defense. While terrestrial phytochemicals have been extensively characterized, a substantial proportion of marine flora remains unexplored, indicating that the chemical diversity of marine ecosystems is still vastly underappreciated.

Beyond their chemical novelty, marine compounds offer distinct mechanistic advantages. Several classes of marine-derived molecules—such as polyphenols, sulfated polysaccharides, carotenoids, and multimineral complexes—modulate conserved inflammatory pathways including NF-κB, MAPK, JAK/STAT3, and the NLRP3 inflammasome, in some cases with greater potency or structural stability than their terrestrial counterparts [2]. Notably, recent reviews integrating flavonoid and anticancer phytochemical literature highlight that structurally defined marine polyphenols effectively converge on NF-κB/MAPK/caspase signaling and ROS balance, which are central to inflammation-driven degeneration in musculoskeletal tissues [3]. Comparative analyses further suggest that marine phytochemicals demonstrate superior bioavailability, metabolic stability, and reactive oxygen species–scavenging capacity, which may underlie their strong effects on musculoskeletal tissues under inflammatory stress (e.g., oxidative myocyte injury, cytokine-driven cartilage degradation, or suppression of osteoblast activity). Marine-derived peptides and amino acid residues have also been shown to act as active modulators of canonical anti-inflammatory pathways, rather than passive nutrients [4].

Advances in environmentally sustainable extraction technologies, such as supercritical fluid and enzyme-assisted extraction, have further enhanced the purity and functional integrity of these compounds, increasing the feasibility of their therapeutic application [1].

Therefore, in the recent years molecules derived from marine plants became promising candidates for improving the condition of the musculoskeletal system, as they have gained increasing attention for their anti-inflammatory, antioxidant, and regenerative effects.

This review first provides an overview of the inflammatory processes occurring in skeletal muscles, tendons, joints, and bones, along with the conventional therapeutic approaches used in their management. It then focuses on marine plant-derived bioactive compounds that may offer effective and safer alternatives for symptom relief and tissue protection. Although these tissues exhibit unique cellular and molecular features, it is essential to emphasize that musculoskeletal inflammation is a multifactorial and interdependent process, wherein crosstalk among resident and infiltrating immune cells, stromal elements, and the extracellular matrix collectively shapes disease progression and resolution.

In line with this approach, this article presents a narrative review based on expert-driven selection and thematic integration of the literature, rather than a systematic or meta-analytical analysis.

## 2. Methods

This narrative review is based on expert-driven selection and thematic integration of the literature. Relevant studies on marine plant-derived bioactive compounds with anti-inflammatory effects in musculoskeletal tissues were identified through searches in PubMed and MEDLINE up to October 2025, supplemented by key references from selected reviews. Although an initial pool of over 3500 articles was screened, final inclusion was guided by relevance to the narrative focus rather than predefined systematic criteria. Human, animal, and in vitro studies were considered to highlight mechanistic insights and potential therapeutic applications. Additional articles were incorporated throughout the writing process to ensure comprehensive coverage of important developments and emerging trends in the field.

## 3. Inflammation

Inflammation represents a double-edged sword of the immune system—an indispensable protective mechanism, yet one that can become antagonistic when dysregulated. While acute inflammation is a highly coordinated and beneficial response aimed at restoring tissue homeostasis [5], chronic inflammation is invariably pathological and contributes to the progression of numerous diseases [6].

Acute inflammation is characterized by five classical signs: redness (rubor) and heat (calor) due to increased blood flow; swelling (tumor) caused by fluid accumulation; pain (dolor) mediated by cytokines and chemokines to limit voluntary movement of the affected area; and loss of function (functio laesa), which reflects the combined effects of pain and swelling and helps protect the injured tissue from further harm.

The inflammatory process proceeds through two principal phases that facilitate tissue regeneration: (1) neutralization of harmful agents and (2) removal of necrotic tissue and apoptotic immune cells. These steps are orchestrated primarily by phagocytic cells, including neutrophils, macrophages, and dendritic cells, under the regulatory influence of T lymphocytes. When inflammation resolves successfully, M2 macrophages promote tissue repair and wound healing by releasing anti-inflammatory cytokines such as IL-10 [7]. However, if the removal of damaged cells remains incomplete, pro-inflammatory M1 macrophages sustain the inflammatory state by secreting cytokines like IL-1, IL-6, and TNF-α [8].

### 3.1. Inflammation of the Musculoskeletal System

**Figure 1:**
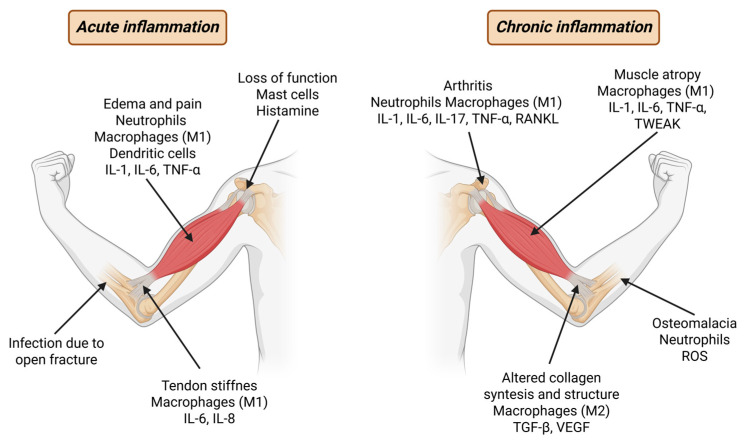
Most common symptoms, mechanisms, cell types and cytokines of acute (**left**) and chronic (**right**) inflammation.

Inflammation of the musculoskeletal system represents a prevalent and clinically significant process that underlies a wide spectrum of acute and chronic conditions, as illustrated in Figure 1. The localization and magnitude of the inflammatory response are determined by the nature, intensity, and duration of the inciting stimulus. Pathogenic microorganisms, chemical agents, and physical insults can elicit localized inflammatory reactions within the components of the musculoskeletal apparatus [9]. Under more severe or systemic exposure, however, inflammation can spread to adjacent tissues or organs, leading to complex and overlapping pathophysiological manifestations.

Based on the anatomical proximity and physiological interdependence of the musculoskeletal system, the homeostasis of muscle, bone, and joint tissues is tightly co-regulated. Inflammatory mediators produced during muscle or tendon injury—including cytokines, prostaglandins, and reactive oxygen species—can reach adjacent bone and joint structures through the bloodstream, disrupting osteoblast–osteoclast balance and promoting synovial inflammation. Conversely, bone-derived factors such as osteokines and joint-derived pro-inflammatory mediators can impair muscle regeneration and contractile function, creating a self-amplifying cycle of musculoskeletal dysfunction. Several marine-derived anti-inflammatory compounds, have been shown to attenuate these processes by reducing cytokine release, improving mitochondrial function in muscle, suppressing osteoclast activation, and limiting synovial inflammation, thereby contributing to the restoration of musculoskeletal homeostasis [10,11].

Damage to skeletal muscle fibers and/or tendons triggers a localized inflammatory response essential for tissue repair but potentially harmful if excessive or prolonged. In skeletal muscle, injury may range from minor microtears to severe inflammation-induced fascial hypertension, where elevated compartmental pressure compromises perfusion and leads to ischemic necrosis. Neutrophil granulocytes dominate the early inflammatory phase, with their proliferation and mobilization controlled by granulocyte colony-stimulating factor (G-CSF) and chemokines such as CXCL12 and CXCL2 [12,13]. Lineage-specific transcription factors, including GFI-1, PU.1, and members of the C/EBP family, guide the differentiation of hematopoietic stem cells toward granulocytic or monocytic fates [14]. Regulation of neutrophil degranulation through intracellular calcium signaling and G protein–coupled receptor pathways, as well as modulation of their interactions with macrophages, T helper cells, and endothelial cells, is critical to prevent secondary tissue injury.

In tendons, persistent inflammation similarly disrupts the balance between pro-inflammatory (M1) and anti-inflammatory (M2) macrophages, impairing extracellular matrix remodeling and reducing collagen synthesis. Over time, this leads to disorganized and weakened collagen fibrils, increased risk of rupture or re-rupture, and excessive scar tissue formation that diminishes musculoskeletal performance [15,16,17]. Hence, coordinated regulation of neutrophil activity, macrophage polarization, and cytokine-mediated signaling is fundamental for the proper resolution of inflammation and restoration of structural and functional integrity in muscle–tendon units.

Bone and joint tissues are dynamic, functionally integrated structures that exhibit distinct yet interrelated inflammatory responses. Bone continuously remodels in response to gravitational forces and mechanical loading, maintaining structural integrity through tightly regulated osteoclastic and osteoblastic activity [18]. Primary inflammation of bone tissue (osteomyelitis) is relatively rare and typically arises from microbial infection, either via hematogenous dissemination under septic or immunodeficient conditions, or direct invasion following open fractures. Clinically, such inflammation manifests as pain, swelling, erythema, and localized heat over the affected site. Chronic or systemic inflammation, however, can disrupt bone metabolism more broadly. Persistent activation of immune pathways—mediated by stress hormones such as cortisol and extracellular acidification resulting from reactive oxygen species—can impair mineralization and contribute to osteomalacia and bone fragility [19,20].

In contrast, inflammation of the joints (arthritis) represents a multifactorial pathology with diverse etiologies. Joint inflammation may result from mechanical trauma, degenerative processes (osteoarthritis), infectious agents (septic arthritis), metabolic disorders such as gout, or autoimmune mechanisms [21,22,23]. When joint inflammation occurs as part of metabolic or autoimmune disease, it is classified as inflammatory arthritis—a chronic, progressive condition that can affect single or multiple joints. Inflammation within the synovial membrane initiates a cascade of tissue damage: infiltration of immune cells compromises synovial integrity, reduces synovial fluid production, and promotes degradation of cartilage and subchondral bone. The complex and enclosed architecture of joints limits the diffusion of therapeutic agents, posing challenges for effective anti-inflammatory treatment. Moreover, the coexistence of mechanical, infectious, metabolic, and immune factors within the same joint microenvironment complicates both diagnosis and targeted intervention.

### 3.2. Conventional Pharmacological Anti-Inflammatory Agents

Nonsteroidal anti-inflammatory drugs (NSAIDs) are among the most widely used agents for managing pain, fever, and inflammation. Their mechanism of action is primarily based on the inhibition of cyclooxygenase (COX) enzymes [24], which catalyse the conversion of arachidonic acid into prostaglandins [25]. Two main isoforms exist: COX-1, a constitutive enzyme involved in homeostatic functions, and COX-2, an inducible enzyme upregulated during inflammation [26]. Inhibition of these enzymes underlies the therapeutic effects of NSAIDs but also explains their adverse effects, including gastrointestinal, cardiovascular, hepatic, and renal toxicity [27], as well as disturbances in haemostasis [28].

Over recent decades, numerous synthetic NSAIDs have been developed and optimized. Classical nonselective COX inhibitors include paracetamol, ibuprofen, naproxen, acetylsalicylic acid and diclofenac [29], whereas celecoxib and etoricoxib selectively inhibit COX-2 [30]. COX-2 selectivity reduces the risk of gastrointestinal ulceration but increases cardiovascular risk, necessitating individualized therapy based on comorbidities and patient profile [31].

NSAIDs are indicated in a broad spectrum of disorders, from acute pain and fever to chronic conditions such as osteoarthritis (OA), where they remain first-line agents for symptom control. NSAIDs can be administered via multiple systemic and local routes, which influence both efficacy and safety. Systemic administration—including oral tablets, capsules, suspensions, and parenteral intravenous or intramuscular injections—ensures distribution throughout the body, making it suitable for generalized pain, fever, or polyarticular inflammation [32]. In contrast, topical administration via gels, creams, patches, or transdermal therapeutic systems allows localized delivery to periarticular tissues, achieving high local concentrations while minimizing systemic exposure and associated adverse effects [33]. The choice of route depends on the clinical indication, required dosage, patient comorbidities, and adherence considerations [34].

Therefore, while conventional NSAIDs remain the cornerstone of pharmacological anti-inflammatory therapy, their use requires careful balancing of efficacy, tolerability, and individual risk factors.

## 4. Marine-Derived Bioactive Compounds as Alternative Anti-Inflammatory Agents

**Figure 2:**
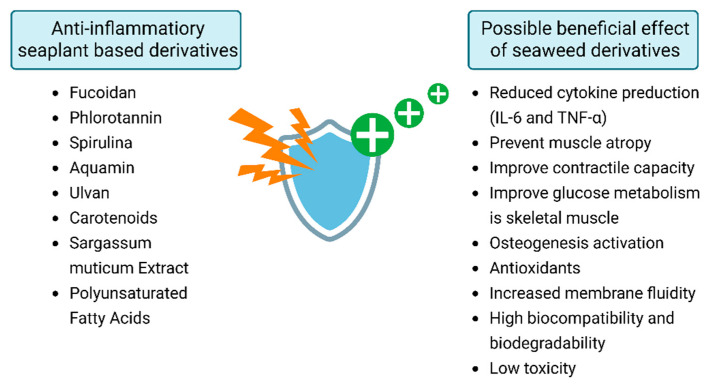
Most frequently used marine-plant-derived substances (**left**) and their potential health benefits (**right**).

Marine-derived bioactive compounds are emerging as promising alternatives or addendums to conventional pharmacological anti-inflammatory therapies. Systemic administration of these compounds has been shown to modulate metabolic and inflammatory pathways relevant to muscle, joint, and bone health, as summarized in Figure 2.

Dietary supplements containing marine plant-derived extracts, particularly from edible brown seaweeds, are widely available and exhibit anti-inflammatory, antioxidant, antimicrobial, and anti-tumor activities [3,35]. Oral intake is generally considered safe, although potential risks include elevated heavy metal content, high iodine levels and arsenic accumulation [36]. Despite these concerns, the incidence and severity of adverse effects are lower than those associated with chronic NSAID use, supporting their potential integration into musculoskeletal inflammation management, including osteoarthritis. Also, the reduction in ROS and cytokine production can limit the unnecessary damage of tissue, prevent drastic metabolic alterations throughout inflammation and speed up regeneration and functional recovery.

Topical administration of marine-derived compounds represents an underexplored but promising approach. Parenteral and transdermal formulations, particularly of fucoidan and marine polyphenols, have demonstrated favorable pharmacokinetics with high local concentrations [37]. Hydrophilic compounds such as fucoidan can be incorporated into hydrophobic carriers—including paraffin- or vaseline-based creams, PEGylated nanoparticles, liposomes, and hydrogels—allowing controlled or extended release [38]. These formulations enable penetration into peri- and intraarticular tissues while maintaining therapeutic plasma levels, with additional benefits such as minimal systemic exposure, enhanced local bioavailability, and increased patient adherence. Recent studies also highlight the versatility of hydrogels and nanoencapsulation in delivering fucoidan, which may further extend its anti-inflammatory and potential anti-cancer effects [39,40].

Overall, both practitioners and patients favor topical delivery in musculoskeletal inflammation due to reduced systemic toxicity, higher local concentrations, and improved compliance. Compared with topical delivery, systemic anti-inflammatory treatments pose a greater risk of masking the symptoms of unrelated or concomitant systemic diseases—for instance, infections—potentially delaying accurate diagnosis and appropriate intervention.

Therefore, comparative evaluation of systemic versus local administration is essential to elucidate differences in pharmacokinetics and pharmacodynamics, optimize therapeutic targeting, and facilitate clinical translation for musculoskeletal disorders. So, while the field remains in its early stages, these strategies hold substantial promise for augmenting conventional therapies and improving outcomes in inflammatory musculoskeletal conditions.

In the following sections, we provide a comprehensive overview of studies examining the anti-inflammatory effects of marine plant-derived bioactive compounds in the musculoskeletal system, with a focus on skeletal muscle, tendons, joints, and bone. This synthesis aims to highlight the therapeutic potential, mechanisms of action, and possible administration strategies of these natural agents in managing musculoskeletal inflammation, with the relevant data summarized in Table 1.

### 4.1. Jack of All Trades: Fucoidan, a Multifunctional Marine-Derived Polysaccharide

Fucoidan, a sulphated heteropolysaccharide primarily composed of L-fucose and sulphate esters, is abundant in brown algae such as *Undaria pinnatifida*. Yang and his colleagues investigated whether fucoidan could improve voluntary exercise capacity, muscle function, and gut microbiome composition in mice maintained on standard or high-fat diets [41]. Mice received fucoidan at 400 mg/kg/day via a calorie-free gel for 10 weeks, ensuring voluntary oral intake. Key endpoints included running distance (wheel activity), muscle mass, grip strength, and mRNA expression of oxidative and mitochondrial genes (COX4, MYH1, PGC-1α, PPAR-γ, IGF1) assessed by RT-qPCR. In mice fed a standard diet, fucoidan increased daily running distance by approximately 25%, muscle mass by 10%, and upregulated oxidative metabolism–related genes. Smaller yet significant improvements were observed under high-fat diet conditions. Microbiome profiling revealed increases in Bacteroides/Bacteroidetes ratio—changes typically associated with improved metabolic health. The authors attributed enhanced performance to both intrinsic muscle remodeling (fiber type transition and mitochondrial biogenesis) and microbiome-mediated metabolic modulation.

These findings are consistent with earlier data by Chen and his co-workers, who demonstrated that oral fucoidan (310–620 mg/kg/day for 21 days) enhanced grip strength and swimming endurance in mice while reducing serum lactate, ammonia, and blood urea nitrogen after exercise without histopathological alterations [42]. Similarly, McBean reported that fucoidan increased muscle fiber cross-sectional area and contractile strength at 400 mg/kg/day [43].

In humans, McFadden and his colleagues administered 1 g/day of fucoidan to healthy adults for 12 days [44]. Although physical performance parameters remained unchanged, plasma IL-6 and IL-10 levels increased post-exercise, suggesting modulation of inflammatory responses during recovery.

Li and his co-workers examined the positive effects of high-purity fucoidan (MW 288.3 kDa, 97.3% purity) in a type 2 diabetes mellitus (T2DM) model [45]. Streptozotocin-induced diabetic rats exhibited hyperglycemia, muscle atrophy, and reduced grip strength, all of which were ameliorated by fucoidan supplementation. Treatment increased fiber cross-sectional area and decreased expression of the atrogenes MuRF-1 and Atrogin-1. Mechanistically, Western blot and kinase assays demonstrated enhanced PI3K/Akt/mTOR/p70S6K signaling and reduced MAPK-driven catabolic activity. In in vitro palmitic acid–treated C2C12 myotubes, fucoidan restored Akt phosphorylation, reduced ROS accumulation, and normalized glucose metabolism gene expression.

Together, these findings suggest that fucoidan counteracts metabolic and inflammatory drivers of muscle loss, supporting its potential utility against sarcopenia and T2DM-associated muscle atrophy.

In the context of bone biology, Nielsen addressed the growing clinical demand for effective bone substitutes and highlighted the promising potential of marine-derived biomaterials in bone tissue engineering [46,47,48]. Using a validated critical-size defect model in seven female sheep—a species exhibiting bone architecture analogous to humans. They placed titanium implants bilaterally in the distal femurs, surrounded by a concentric 2 mm gap filled with hydroxyapatite (HA) coated with low-molecular-weight (LMW) fucoidan [49]. After 12 weeks, mechanical push-out testing, micro-computed tomography (micro-CT), immunohistochemistry, and histomorphometry demonstrated pronounced new bone formation in all HA/fucoidan (HA/FUC) groups, with fixation strength comparable to that of allograft controls. Microarchitectural indices, including bone volume fraction and trabecular thickness, were improved, supporting the role of fucoidan in promoting osteogenesis and implant integration. The authors emphasized the importance of LMW fucoidan, which exhibits pro-angiogenic and osteogenic properties, in contrast to high-molecular-weight variants that may exert anti-angiogenic effects [50,51]. Despite limited sample size and challenges in quantifying angiogenesis, the findings underscore the potential of fucoidan as a biomaterial component for orthopedic implant fixation and bone regeneration.

Further evidence was provided by Liu and his colleagues, who evaluated fucoidan (FPS) in a rat model of chronic kidney disease–mineral and bone disorder (CKD-MBD)—a syndrome characterized by renal osteodystrophy, impaired mineral metabolism, and elevated fracture risk [52]. The authors induced CKD-MBD via adenine administration combined with uninephrectomy and treated rats with FPS or calcitriol (vitamin D analogue) for 21 days. FPS ameliorated renal dysfunction, improved serum calcium-phosphorus balance, and enhanced bone mineral density and trabecular microarchitecture as assessed by micro-CT. Mechanistically, FPS modulated the FGF23–Klotho axis, restoring phosphate regulatory signaling disrupted in CKD [53]. These effects were comparable or superior to calcitriol but without the associated vascular calcification risk, indicating that fucoidan exerts multi-targeted protective actions on kidney and bone through metabolic and endocrine modulation [54].

Regarding joint health, several in vitro and in vivo investigations have elucidated the anti-inflammatory and immunomodulatory properties of fucoidan. Fucoidan significantly reduced nitric oxide (NO) and prostaglandin E_2_ (PGE_2_) production in LPS-stimulated RAW 264.7 macrophages by downregulating iNOS and COX-2, while suppressing pro-inflammatory cytokines (IL-1β, TNF-α, IL-6) through NF-κB and MAPK pathway inhibition in a dose-dependent manner [55]. Similarly, fucoidan supplementation attenuated inflammation and improved clinical outcomes in complete Freund’s adjuvant (CFA)-induced arthritis in Wistar rats by lowering pro-inflammatory and enhancing anti-inflammatory cytokine expression, despite lacking direct radical-scavenging activity [56].

In an osteoarthritis model, Chiang and his co-workers tested an oligo-fucoidan-based formulation (>30% oligo-fucoidan) in monosodium iodoacetate (MIA)-induced osteoarthritis [57]. The treatment modulated p38 MAPK signaling, reduced COX-2 and iNOS levels, and mitigated cartilage degradation and joint inflammation. These findings collectively highlight fucoidan’s potential to alleviate joint inflammation and protect cartilage integrity via suppression of pro-inflammatory signaling and oxidative stress.

Taken together, data across bone, muscle, and joint models demonstrate fucoidan’s broad bioactivity—ranging from osteogenic and angiogenic enhancement to anti-inflammatory and metabolic regulation—supporting its translational promise as a multifunctional marine-derived therapeutic and biomaterial component in musculoskeletal health.

### 4.2. Marine-Derived Polyphenols as Anti-Inflammatory Agents

Phlorotannins are polyphenolic compounds unique to brown algae, including *Ecklonia cava*, *Ishige okamurae*, and *Sargassum horneri*. Structurally, they consist of oligomers or polymers of phloroglucinol often bound to the algal cell wall matrix. Numerous studies have demonstrated their ability to inhibit key inflammatory signalling pathways such as NF-κB, MAPK (ERK, JNK, p38), JAK/STAT3 [58], and the NLRP3 inflammasome (containing Toll-like receptor 4), leading to downregulation of TNF-α, IL-1β, IL-6, and other proinflammatory mediators in in vitro and in vivo models [59,60]. The relationship between the anti-inflammatory effect and TLR-mediated NF-κB/MAPK activation is particularly relevant, as phlorotannins can modulate upstream pattern-recognition receptors including TLRs and scavenger receptors, thus influencing the initiation of the inflammatory cascade, however, further studies are needed to verify this hypothesis.

In Kim’s laboratory five brown algae extracts were examined—*Ishige okamurae*, *Ecklonia cava*, *Hizikia fusiforme*, *Myelophcus caespitosus*, and *Sargassum horneri*—for their dual effects on inflammation and muscle anabolism [61]. All extracts reduced nitric oxide production in LPS-stimulated RAW 264.7 macrophages without cytotoxicity, while *Ishige okamurae* extract uniquely enhanced C2C12 myoblast proliferation. Fractionation and chemical characterization identified two active phlorotannins, diphlorethohydroxycarmalol (DPHC) and ishophloroglucin A (IPA). Molecular docking to the TNF-α trimer (PDB 2AZ5) revealed DPHC as the most stable binder. In TNF-α–stimulated C2C12 myotubes, DPHC suppressed MuRF-1 and Atrogin-1 expression, reduced NF-κB p65 nuclear translocation, and inhibited MAPK phosphorylation (ERK, JNK, p38), demonstrating both anti-catabolic and pro-anabolic effects.

Additional studies confirmed that other brown algae polyphenols, such as dieckol and phloroglucinol-bieckol from *Ecklonia cava*, also enhance protein synthesis and improve contractile function in C2C12 cells and dexamethasone-induced atrophy models via NF-κB/MAPK modulation [62]. Similarly, Kim and his co-workers showed that mixed extracts containing polyphenols and polysaccharides from *Undaria pinnatifida*, *Codium fragile*, and *Gracilaria gracilis* (formerly *Gracilaria verrucosa*) improved glucose utilization and reduced inflammatory markers in C2C12 myotubes.

In bone metabolism, Kim and Park investigated the osteoprotective effects of *Ishige sinicola* extract (ISE), a phlorotannin-rich preparation from a brown alga native to East Asia, in the context of postmenopausal osteoporosis [63]. Osteoporosis—marked by low bone mass and microarchitectural deterioration due to estrogen deficiency—increases fracture risk after menopause [64]. Using an ovariectomized (OVX) rat model, a well-established analogue of estrogen-deficient bone loss, the authors administered ISE at 20 or 200 mg/kg for six weeks, with estradiol as a positive control [65]. Micro-CT and histological analyses revealed that ISE treatment significantly improved bone mineral density, trabecular thickness, and trabecular number while reducing trabecular separation. In vitro, ISE inhibited RANKL-induced osteoclast differentiation in RAW 264.7 macrophages, lowering the number of TRAP-positive multinucleated cells and suppressing osteoclastogenic transcription factors NFATc1 and c-Fos, along with TRAP, c-Src, and cathepsin K protein expression. These findings indicate that ISE counteracts bone resorption by downregulating the NFATc1/c-Fos pathway and promoting bone formation, aligning with previous evidence on the anti-osteoclastic and osteogenic potential of brown algae–derived compounds such as fucoidan [66]. Collectively, ISE shows promise as a nutraceutical or adjunctive therapeutic for postmenopausal osteoporosis.

Regarding joint physiology, phlorotannins exert potent anti-inflammatory and antioxidant activities, primarily by modulating the canonical NF-κB-dependent signaling and inhibiting the production of TNF-α, IL-6, and IL-1β [67,68]. In vitro experiments have also demonstrated that phlorotannin-rich extracts attenuate allergic and inflammatory responses by inhibiting hyaluronidase, an enzyme involved in inflammatory exudation, and reduce the synthesis of nitric oxide (NO) and prostaglandins such as PGE_2_ by downregulating iNOS and COX-2 expression [69,70]. Furthermore, phlorotannins have been reported to inhibit matrix metalloproteinases (MMPs), enzymes that mediate extracellular matrix degradation and contribute to cartilage destruction in arthritis [69]. Their antioxidant properties further support chondroprotection by mitigating oxidative stress and limiting free radical formation [67].

Although dietary absorption of phlorotannins is limited, ongoing research aims to improve their bioavailability and formulation stability to enhance clinical efficacy [68].

Recent studies in the broader polyphenol field indicate that the biological activity of flavonoids can be substantially enhanced through advanced delivery systems. A recent review highlights how liposomes, polymeric nanoparticles, solid–lipid nanoparticles, and mixed micelles improve stability, tissue distribution, and target specificity of flavonoids by protecting them from rapid degradation and facilitating uptake in oxidatively stressed or inflamed tissues [3]. While most of these studies focus on terrestrial flavonoids, the principles are directly applicable to marine-derived polyphenols. Phlorotannins share similar limitations in solubility, stability, and cellular penetration, suggesting that nanoformulation-based strategies could enhance their bioavailability and accumulation within inflamed musculoskeletal tissues. Combining such delivery approaches with the known NF-κB, MAPK, and JAK/STAT-targeting effects of phlorotannins may therefore represent a promising strategy to boost their anti-inflammatory potential and broaden their therapeutic applicability.

Taken together, these findings suggest that phlorotannins represent promising marine-derived polyphenols capable of modulating bone remodeling, suppressing joint inflammation, and protecting cartilage integrity through their combined antioxidant, anti-inflammatory, and anti-catabolic actions.

### 4.3. Spirulina

Spirulina (*Arthrospira platensis* and *A. maxima*), a cyanobacterial biomass rich in proteins (≈64 g/100 g dry weight), vitamins, minerals, β-carotene, and the pigment–protein complex phycocyanin, has been extensively studied for its antioxidant and anti-inflammatory properties [71,72].

Previous studies demonstrated spirulina’s capacity to mitigate exercise-induced oxidative stress and inflammation in muscles of both animal models and humans [73,74,75]. In a randomized, double-blind, placebo-controlled trial, Chaouachi investigated the effects of *A. platensis* supplementation on oxidative stress, inflammation, and muscle injury in elite Rugby Union players [76]. Seventeen athletes consumed 5.7 g/day of spirulina or an isocaloric/isoproteic placebo for seven weeks during the competitive season. Using the Yo-Yo Intermittent Recovery Test 2 (YYIRT-2) to elicit high oxidative and inflammatory stress, the authors measured biomarkers before, immediately after, and 24 h post-exercise. In the placebo group, plasma levels of F_2_-isoprostanes, C-reactive protein (CRP), and creatine kinase (CK) rose significantly after exertion, while spirulina supplementation prevented these increases and accelerated post-exercise recovery. The protective effects were attributed to phycocyanin and β-carotene, which scavenge free radicals, inhibit lipid peroxidation, and downregulate iNOS, COX-2, and proinflammatory cytokines. Oxidative stress markers—including superoxide dismutase, glutathione peroxidase activity, etc.—consistently indicate lowered ROS production following spirulina treatment. Importantly, phycocyanin exerts its anti-inflammatory effect through both ROS-dependent and ROS-independent pathways: reduced oxidative stress limits the activation of redox-sensitive inflammatory cascades, while phycocyanin also directly suppresses cytokine signaling, leading to decreased TNF-α, IL-1β, and IL-6 release. Together, these dual actions contribute to its protective role in musculoskeletal tissues. These results support spirulina’s potential to attenuate oxidative and inflammatory muscle damage, thereby preserving recovery and performance in athletes under high physical load.

Pharmacological treatment of diabetic bone fragility is complicated by adverse drug effects—metformin, for example, has been linked to decreased bone formation and vitamin B_12_ deficiency, which may enhance osteoclastogenesis [77,78]. Ogechi Ekeuku investigated spirulina’s effects on diabetic osteopenia in female Sprague–Dawley rats [79]. Diabetes was induced using streptozotocin (50 mg/kg), and diabetic animals were treated for 12 weeks with metformin (300 mg/kg), spirulina (300 mg/kg), or their combination. Compared to diabetic and metformin-only groups, spirulina markedly improved bone mineral density, trabecular connectivity, and mechanical strength (maximum force, stress, and elastic modulus). Histological analyses showed restored trabecular structure and increased osteoblast and osteocyte numbers, paralleled by upregulation of osteocalcin (OCN) expression. The authors linked these effects to spirulina’s antioxidant, hypoglycemic, and micronutrient-stabilizing properties—chromium-mediated enhancement of insulin signaling, β-carotene–induced hepatoprotection, and prevention of vitamin B_12_ depletion. Overall, spirulina outperformed metformin in restoring bone structural integrity and strength, highlighting its potential to counteract diabetes-related skeletal fragility.

Clinical and experimental data also support spirulina’s systemic anti-inflammatory role relevant to joint pathology. A recent meta-analysis encompassing 22 trials found that spirulina supplementation significantly reduced circulating TNF-α, IL-6, and CRP levels, while oxidative stress markers such as malondialdehyde decreased and total antioxidant capacity increased [80]. Although IL-2 levels remained unchanged, these findings collectively indicate potent immunomodulatory and redox-balancing effects. Given the central role of oxidative stress and low-grade inflammation in the pathogenesis of osteoarthritis and related joint disorders, spirulina represents a promising adjuvant candidate for maintaining joint homeostasis and attenuating chronic inflammatory progression.

Together, these findings underscore spirulina’s multifunctional protective role across muscle, bone, and joint tissues through convergent antioxidant, anti-inflammatory, and metabolic pathways, supporting its translational potential as a nutraceutical intervention in musculoskeletal health.

### 4.4. Aquamin

Aquamin is a natural, calcium-rich multimineral complex derived from the red marine algae *Lithothamnion*. It contains bioavailable forms of calcium (aragonite, vaterite, and calcite), magnesium, strontium, and over 70 trace elements essential for bone and connective tissue metabolism. The calcium-sensing receptor (CaSR) may be activated by the increased extracellular calcium from Aquamin, which could reduce parathyroid hormone secretion and be beneficial for osteogenesis [81]. Additionally, trace minerals such as magnesium and strontium might modulate key osteogenic pathways, including Wnt/β-catenin signaling and the RANKL/OPG balance, supporting osteoblast differentiation and limiting osteoclast activity. Its unique mineral composition has been shown to enhance biomechanical performance and exert osteogenic, anti-inflammatory, and antioxidative effects relevant to bone and joint health. Despite these, no experimental or clinical data are currently available regarding the use of Aquamin in inflammatory muscle disorders or muscle regeneration.

Recognizing the limitations of conventional bone graft substitutes, Brennan developed a collagen–glycosaminoglycan (GAG) scaffold incorporating Aquamin at 100 wt% and 500 wt% relative to collagen [82]. The inclusion of Aquamin significantly increased scaffold stiffness while maintaining optimal porosity and permeability for cellular infiltration and nutrient diffusion. Release kinetics showed an initial burst followed by sustained mineral ion release, favoring prolonged osteogenic stimulation. When MC3T3-E1 osteoblasts were cultured on these scaffolds, alkaline phosphatase (ALP), osteopontin, and osteocalcin expression were upregulated, and mineralization was confirmed via von Kossa and Alizarin Red staining. Thus, Aquamin enhanced both the mechanical and biological performance of the scaffold, demonstrating potential as a natural, cell-free, osteostimulative biomaterial for bone regeneration.

In a follow-up in vivo study of Brennan an ovariectomized (OVX) rat model of postmenopausal osteoporosis was employed to compare Aquamin with calcium carbonate supplementation [83]. Over 20 weeks, analyses by microCT, FTIR, and nanoindentation revealed that Aquamin significantly preserved trabecular architecture, hydroxyapatite mineral density, and mechanical strength, outperforming calcium carbonate. This was attributed to its superior bioavailability and synergistic mineral composition [84,85].

Complementary in vitro research by O’Gorman confirmed that Aquamin directly promotes osteoblast differentiation and mineralization in MC3T3-E1 preosteoblastic cells [86]. ALP activity increased, and enhanced extracellular matrix mineralization was observed by day 28. Importantly, the effect could not be replicated by calcium alone, implying synergistic contributions from trace elements such as magnesium, zinc, and strontium. Building on these findings, Widaa and his colleagues demonstrated that combining Aquamin with vitamin D3 amplified osteogenic outcomes—ALP activity and mineralization were significantly higher than with either treatment alone, suggesting that Aquamin provides essential cofactors for osteoblast function while vitamin D_3_ upregulates osteogenic gene expression [87,88]. These data collectively position Aquamin as a multifunctional osteonutrient with therapeutic potential in osteoporosis and bone tissue engineering.

Aquamin F, the standard multimineral formulation, has shown clinical efficacy in managing osteoarthritis (OA). In a human intervention study, Aquamin improved joint comfort and mobility in individuals with moderate-to-severe OA without adverse effects [89]. As Aquamin contains numerous bioactive minerals, isolating a single “active ingredient” is impractical. Instead, its collective composition—rich in calcium, magnesium, and trace elements with documented anti-inflammatory and antioxidant properties—appears to act synergistically to reduce cartilage inflammation and oxidative stress. These findings suggest that Aquamin could serve as a safe nutraceutical addition to conventional OA therapies, potentially reducing dependence on NSAIDs while supporting joint function and integrity.

Overall, evidence from in vitro, in vivo, and clinical studies highlights Aquamin as a promising marine-derived multimineral complex that enhances osteogenesis, preserves bone microarchitecture, and alleviates joint inflammation. Its biocompatibility and synergistic mineral profile make it a strong candidate for applications in bone regeneration, osteoporosis prevention, and the nutritional management of degenerative joint diseases.

Although direct mechanistic studies on Aquamin remain limited, the observed enhancement of osteoblast differentiation, mineralization, and bone strength in vitro and in vivo is consistent with modulation of these pathways. Activation of CaSR by extracellular calcium, together with trace mineral–mediated effects on Wnt/β-catenin signaling and the RANKL/OPG balance, likely contributes to the improved osteogenic outcomes and reduced osteoclast activity reported across studies. This integrated perspective provides a mechanistic basis for Aquamin’s multifunctional effects on bone and joint health.

### 4.5. Additional Marine Plant-Derived Bioactives of Musculoskeletal Interest

Beyond fucoidan, phlorotannins, spirulina, and Aquamin, several additional bioactive compounds derived from marine algae have demonstrated promising anti-inflammatory and antioxidative properties relevant to musculoskeletal health. However, current evidence is fragmentary, with most studies addressing isolated tissues or cellular mechanisms rather than the entire musculoskeletal system.

#### 4.5.1. Ulvans

Ulvans are sulphated polysaccharides extracted from the cell wall of green macroalgae of the genus Ulva (Chlorophyta), comprising between 88 and 400 recognized species [90,91]. Chemically, ulvans consist primarily of rhamnose, uronic acids (D-glucuronic and L-iduronic acid), and xylose, conferring a structural resemblance to mammalian glycosaminoglycans such as heparan sulphate, chondroitin sulphate, and dermatan sulphate. This similarity, coupled with their high biocompatibility, biodegradability, and low toxicity, has drawn attention to their potential in biomedical applications including wound healing and tissue regeneration [92,93].

Mechanistically, ulvans exert anti-inflammatory activity by modulating key intracellular signaling cascades, thereby inhibiting apoptosis, suppressing pro-inflammatory enzymes, and regulating transcription factors involved in inflammatory gene expression. Reported molecular targets include COX-2, iNOS, NF-κB, MAPK, nrf-2, PPARs, Toll-like receptors, TNF-α, and various interleukins [92,94,95,96]. These properties suggest ulvans as potential candidates for biomaterial development aimed at musculoskeletal inflammation modulation.

The anti-inflammatory effect of ulvans is influenced by their structural characteristics, including the degree of sulfation and molecular branching, which modulate their interactions with cell surface receptors and signaling proteins. Highly sulfated and branched ulvans have been shown to more effectively inhibit NF-κB and MAPK activation, reduce proinflammatory cytokine release, and enhance Nrf2-mediated antioxidant responses in vitro [97,98]. This structure-function relationship provides a mechanistic basis for their potential application in musculoskeletal inflammation and tissue regeneration.

**Table 1:** Most important characteristics of the marine plant derivatives reviewed in this article.

**In Vitro**			
**Plant Source**	**Model Used**	**Effect**	**Reference**
Brown alga (*Ishige okamurae*)	RAW 264.7 macrophages, C2C12 myoblasts	Inhibited NO production, promoted myoblast proliferation	[61]
Brown alga (*Turbinaria decurrens*)	RAW 264.7 macrophages	Strong NO inhibition	[56]
Brown algae mix (*Undaria pinnatifida* & *Fucus vesiculosus*)	RAW 264.7 macrophages	Reduced iNOS expression, anti-inflammatory	[57]
Brown alga (*Ecklonia cava*)	C2C12 myoblasts	Promoted muscle growth via Smad & IGF-1 pathways	[62]
Brown alga (*Ecklonia cava*)	HT1080, human fibro sarcoma and human dermal fibroblast cell lines	Inhibited matrix metalloproteinases	[69]
Brown algae (*Ishige sinicola*)	RAW264.7 murine macrophages	Inhibition of osteoclast formation	[63]
Spirulina (*Arthrospira*)	C2C12 myotubes	Prevented myotube atrophy, reduced Atrogin-1/MuRF1, improved glucose metabolism via PI3K/Akt	[45]
Red algae (*Lithothamnion calcareum*)	Osteoblast cell culture	Enhanced bone mineralization	[86]
Red algae (*Lithothamnion calcareum*)	Osteoblast cells	Enhanced bone mineralization, increased osteogenesis	[83]
**In Vivo—Systemic**			
**Plant Source**	**Model Used**	**Effect**	**Reference**
Brown alga (*Ishige okamurae*)	TNF-α-induced inflammatory myopathy in mice/cell-derived model	Downregulated MuRF-1, MAFbx via NF-κB/MAPK	[61]
Brown alga (*Turbinaria decurrens*)	CFA-induced arthritis in Wistar rats	Reduced inflammation, paw edema, modulated cytokines	[56]
Brown algae mix (*Undaria pinnatifida* & *Fucus vesiculosus*)	MIA-induced osteoarthritis in rats	Reduced joint swelling, improved cartilage via p38/iNOS/COX-2 suppression	[57]
Brown alga (*Ecklonia cava*)	C57BL/6J mouse model	Increased muscle mass and activity	[62]
Red algae (*Lithothamnion corallioides*)	Human osteoarthritis patients	Improved WOMAC scores, walking distance, reduced NSAID use	[89]
Red algae (*Lithothamnion calcareum*)	Mice on Western-style diet	Preserved bone structure/function	[86]
Red algae (*Lithothamnion calcareum*)	Ovariectomised Wistar rat model	Preserved bone structure, enhanced osteogenesis	[83]
Fucoidan (*Laminaria japonica*)	ICR strain mouse model	Increased muscle strength and performance, reduced post-exercise inflammation	[42]
Brown algae (*Ishige sinicola*)	Ovariectomy induced mouse model	improved the trabecular bone structure, bone biomechanical properties, and bone mineralization degree	[63]
Spirulina (*Arthrospira*)	Streptozotocin-induced type 2 diabetic female Sprague Dawley rats	Improved trabecular bone microarchitecture, increased vitamin D, lowered serum Ca/P, increased bone strength/stiffness, increased osteocalcin	[79]
Fucoidan (*Laminaria japonica*)	Adenine-induced CKD–MBD rats	Improved bone mineral density & microarchitecture, corrected Ca/P metabolism, modulated FGF23–Klotho axis	[52]
Spirulina (*Arthrospira platensis*)	Human trial with exhaustive exercise (Yo Yo IR2 test) model	Reduced exercise-induced lipid peroxidation, CRP, CK; accelerated recovery	[76]
Brown algae (*Undaria pinnatifida*, *Fucus vesiculosus*)	C57BL/6 mouse model	Increased muscle size & strength	[43]
Brown algae (*Undaria pinnatifida*, *Fucus vesiculosus*)	Humans exercise recovery model	Reduced inflammation post-exercise	[44]
**In Vivo—Topical**			
**Plant Source**	**Model Used**	**Effect**	**Reference**
Red marine algae (*Lithothamnion*)	In vitro osteoblast culture on collagen–GAG scaffolds	Enhanced osteogenesis, increased mineralization, improved scaffold mechanical properties	[82]
Brown algae (*Fucus serratus* & *Fucus evanescens*)	Sheep bilateral distal femur critical-size defect implant gap model	Bone formation in all HA/Fucoidan groups, similar implant fixation strength to allograft, but less bone volume fraction than allograft	[46]

#### 4.5.2. Carotenoids

Marine algae and microalgae synthesize diverse carotenoids—including astaxanthin, fucoxanthin, and β-carotene—that impart yellow to red pigmentation and exhibit potent antioxidant activity. Their mechanisms of action are multimodal: scavenging singlet oxygen, stabilizing hydroperoxides, quenching free radicals, interrupting auto-oxidation chains, and chelating redox-active metal ions [97,98]. Another major mechanism of carotenoids involves modulation of transcription factors such as Nrf2 and FoxOs, in addition to their antioxidant activity. The Nrf2 pathway is activated under oxidative stress conditions, including inflammation and ischemia. Tamayo et al. [99] reported that in an ischemia–reperfusion muscle model, local release of astaxanthin decreased Nrf2 activity, suggesting a potential role in protecting against ROS-induced inflammation. Forkhead box O (FoxO) proteins promote inflammation by upregulating pro-inflammatory molecules such as TLR4, IL-1β, and TNF-α. Zhiyin et al. found that fucoxanthin mitigated dexamethasone-induced atrophy in C2C12 myotubes by inhibiting FoxO activity via SIRT1-mediated phosphorylation and deacetylation of FoxO3 [100]. These findings indicate that carotenoids can suppress inflammation-associated FoxO activation, complementing their antioxidant effects. These activities contribute to the suppression of oxidative stress, a major driver of inflammation and tissue degeneration in musculoskeletal disorders.

#### 4.5.3. *Sargassum muticum* Extract

The brown alga *Sargassum muticum*, an edible and widely distributed species, has demonstrated pronounced immunomodulatory potential. Extracts of *S. muticum* significantly inhibited the production of key pro-inflammatory cytokines—including IL-12p40, IL-6, and TNF-α—in mouse bone marrow–derived macrophages and dendritic cells. The effect was dose-dependent, with IC_50_ values between 5.31 and 13.79 μg/mL. Mechanistic studies revealed inhibition of ERK1/2 phosphorylation and suppression of activator protein-1 (AP-1) reporter activity, indicating interference with upstream signaling cascades critical for cytokine transcription [101]. These findings suggest a potential therapeutic application of Sargassum-derived compounds in inflammatory joint and soft-tissue pathologies.

#### 4.5.4. Polyunsaturated Fatty Acids (PUFAs)

Three polyunsaturated fatty acids (PUFAs) isolated from the edible brown seaweed *Undaria pinnatifida*—stearidonic acid (SA), eicosapentaenoic acid (EPA), and arachidonic acid (AA)—exhibit differential inflammatory properties. While the omega-3 PUFAs SA and EPA are known to exert anti-inflammatory effects by competing with arachidonic acid metabolism and reducing prostaglandin synthesis, omega-6 AA serves as a pro-inflammatory precursor [102]. The balance of these fatty acids may therefore critically influence musculoskeletal inflammatory processes and tissue repair dynamics.

Altogether, these compounds—ulvans, carotenoids, *Sargassum muticum* extract, and algal-derived PUFAs—represent emerging natural agents with significant anti-inflammatory and antioxidative potential. Nonetheless, most available evidence remains limited to in vitro or early in vivo models, and systematic evaluation of their efficacy across muscle, bone, and joint systems is warranted to establish their therapeutic value in musculoskeletal medicine.

### 4.6. Synergy Between Marine Plant-Based Compounds

Exploring potential synergy between marine plant-derived bioactives is critical for the development of combination nutraceuticals with enhanced therapeutic efficacy.

Recent studies demonstrated that fucoidan + phlorotannin combinations mitigated amyloid-beta-induced cognitive deficits in mice, supported by behavioral tests (Y-maze, water maze) and molecular analyses. Enhanced mitochondrial function, increased antioxidant capacity, and suppressed phosphorylation of MAPK pathway components (Akt, JNK) collectively contributed to stronger anti-inflammatory effects [103].

Fixed combinations of fucose (the main structural component of fucoidan) and phloroglucinol (representative polyphenol) solutions showed increased radical scavenging activity, highlighting how these compounds may mitigate oxidative stress and improve overall therapeutic outcomes [104,105].

Spirulina is a rich source of micro- and macronutrients, particularly iron (28–50 mg/100 g dry weight) and B vitamins (B1, B6, B9), with generally high bioavailability [106,107]. Co-supplementation with vitamin C can further enhance iron absorption, while vitamin B12 supplementation could synergistically improve hematopoiesis and DNA synthesis. Such combinatorial approaches may also support musculoskeletal health, energy metabolism, and oxidative stress defense, illustrating the translational potential of synergistic marine-derived nutraceutical formulations.

Taken together, these findings highlight that rational combinations of marine plant-based compounds can potentiate anti-inflammatory, antioxidant, and regenerative effects, providing a mechanistic rationale for the development of combination nutraceuticals targeting musculoskeletal health. Such approaches may allow for lower individual doses, reduced off-target effects, and improved clinical efficacy, particularly in complex inflammatory or degenerative conditions.

## 5. Conclusions

Marine plant-derived bioactives represent a promising class of agents for the management of musculoskeletal inflammatory conditions, offering high biocompatibility, favorable safety profiles, and potential for both systemic and topical administration. Despite their efficacy, many applications and mechanisms remain underexplored, particularly for topical formulations, highlighting a key avenue for future research.

The discovery of novel, highly specific anti-inflammatory molecules remains a critical goal. Marine-derived bioactive compounds provide a valuable starting point for the development of such candidates, potentially leading to the identification of new receptors, signaling pathways, and finer regulatory mechanisms of inflammation. Among these, fucoidans stand out as particularly promising due to their relatively simple chemical structure, well-defined biochemical reactivity, and demonstrable anti-inflammatory activity. Their mechanistic effects are more amenable to detailed characterization compared with multifactorial extracts such as whole brown algae or Spirulina preparations. However, the safety profile, off-target effects, and pharmacokinetics of these compounds require further investigation. Multi-component extracts, while biologically active, pose additional challenges for clinical translation, including variability in patient response and potential interactions among components.

From an environmental perspective, production of marine-derived bioactives—via extraction from sustainably sourced algal biomass or mineral-rich matrices such as Aquamin—typically involves fewer energy-intensive chemical reactions and generates less hazardous waste than conventional synthetic pharmaceuticals, including NSAIDs [108,109,110]. Nonetheless, the ecological benefit depends on sustainable sourcing, efficient extraction processes, and responsible scale-up.

Collectively, these observations underscore the dual advantage of marine-derived bioactives: they provide both therapeutic potential and mechanistic insight into musculoskeletal inflammation, while offering a potentially reduced ecological footprint. Continued research integrating mechanistic studies, optimized delivery strategies, and translational evaluation will be essential to realize their full clinical potential.

## Data Availability

No new data were created or analyzed in this study. Data sharing is not applicable to this article.
